# Surveillance-based evidence: elimination of schistosomiasis as a public health problem in the Peoples’ Republic of China

**DOI:** 10.1186/s40249-020-00676-5

**Published:** 2020-06-06

**Authors:** Jing Xu, Shi-Zhu Li, Li-Juan Zhang, Robert Bergquist, Hui Dang, Qiang Wang, Shan Lv, Tian-Ping Wang, Dan-Dan Lin, Jian-Bing Liu, Guang-Hui Ren, Kun Yang, Yang Liu, Yi Dong, Shi-Qing Zhang, Xiao-Nong Zhou

**Affiliations:** 1 National Institute of Parasitic Diseases, Chinese Center for Disease Control and Prevention, WHO Collaborating Centre for Tropical Diseases, Chinese Center for Tropical Disease Research, Shanghai, 200025 People’s Republic of China; 2 Geospatial Health, Ingerod 407, S-45494 Brastad, Sweden; 3 Anhui Provincial Institute of Schistosomiasis Control, Hefei, Anhui Province 230061 People’s Republic of China; 4 Jiangxi Provincial Institute of Parasitic Disease, Nanchang, Jiangxi Province 330006 People’s Republic of China; 5 Hubei Provincial Institute of Schistosomiasis Control, Hubei Center for Disease Control, Wuhan, Hubei Province 430079 People’s Republic of China; 6 Hunan Provincial Institute of Schistosomiasis Control, Yueyang, Hunan Province 414000 People’s Republic of China; 7 Jiangsu Provincial Institute of Schistosomiasis Control, Wuxi, Jiangsu Province 214064 People’s Republic of China; 8 Sichuan Center for Disease Control, Chengdu, Sichuan Province 610041 People’s Republic of China; 9 Yunnan Provincial Institute of Endemic Diseases Control and Prevention, Dali, Yunnan Province 671000 People’s Republic of China

**Keywords:** Schistosomiasis, Surveillance, Infection rate, Elimination, China

## Abstract

**Background:**

A steady progress on schistosomiasis control in the Peoples’ Republic of China (P.R. China) was achieved and broadened into the twelve-year medium and long term national plan (MLNP) which marled the implementation of an integrated control strategy across all endemic areas in P.R. China in 2004. To understand the endemic trends of schistosomiasis to assess the effectiveness of an integrated strategy, we conducted an analysis of schistosomiasis surveillance data spanned from 2005 to 2015.

**Methods:**

The schistosomiasis sentinel surveillance data from sentinel sites were collected and analyzed from 2005 to 2015. In these sentinel sites, residents aged 6 years or above were screened annually by indirect hemagglutination assay (IHA), while only antibody positives were followed by stool examination either Kato-katz method (KK) and/or hatching technique (HT). Domestic animals raised in sentinel sites were examined by HT for confirming the infection of schistosomes. Snail investigation was conducted each year through systematic sampling method combined with environmental sampling method. The snails collected from field were tested by microscopic dissection method. The infection rates of schistosomes in residents, domestic animals and snails, as well as the indicators reflecting the snails’ distribution were calculated and analyzed. ANOVA analysis was used to examine the changes of the number of eggs per gram feces in population and Chi-square test was used to examine any change in proportions among groups.

**Results:**

A total of 148 902 residents from sentinel sites attended this study and 631 676 blood samples were examined by IHA test during the 11 covered years. The annual average antibody positive rates presented a significant decrease trends, from 17.48% (95% *CI*: 17.20–17.75%) in 2005 to 5.93% (95% *CI*: 5.71–6.15%) (*χ*^2^ = 8890.47, *P* < 0.001) in 2015. During 2005–2015, the average infection rate of schistosomes in residents declined from 2.07% (95% *CI*: 1.96–2.17%) to 0.13% (95% *CI*: 0.09–0.16%), accompanied by significant decrease of infection intensity in population. In 2015, the stool positives were only found in farmers, fishermen and boatmen with infection rate of 0.16% (95% *CI*: 0.11–0.20%), 0.17% (95% *CI*: 0–0.50%) respectively. The infection rate of schistosomes in domestic animals dropped from 9.42% (538/5711, 95% *CI*: 8.66–10.18%) to 0.08% (2/2360, 95% *CI*: 0–0.20%) from 2005 to 2015. Infections were found in eight species of domestic animals at the beginning of surveillance while only two cattle were infected in 2015. Totally 98 ha of new snail habitats were found, while 94.90% (93/98) distributed in lake and marshland regions. The percentage of frames with snails decreased from 16.96% (56 884/33 5391, 95% *CI*: 16.83–17.09%) in 2005 to 4.28% (18 121/423 755, 95% *CI*: 4.22–4.34%) in 2014, with a slightly increase in 2015. Meanwhile, the infection rate of schistosomes in snails was decreased from 0.26% (663/256 531, 95% *CI*: 0.24–0.28%) to zero during 2005–2015.

**Conclusions:**

The infection rate of schistosomes declined significantly, providing evidence that the goal of the MLNP was achieved. Elimination of schistosomiasis as a public health problem defined as WHO was also reached in P.R. China nationwide. Surveillance-response system should be improved and strengthened to realize the final goal of schistosomiasis elimination.

## Background

Schistosomiasis is a globally distributed disease caused by three major *Schistosoma* species: *S. mansoni, S. haematobium* and *S. japonicum* [1], all depending on intermediate snail hosts to complete their life cycles. Humans get infection when contact with freshwater containing cercaria of schistosomes. In contrast to other schistosome species, *S. japonicum* is a zoonotic species with a wide spectrum of wild and domestic animals serving as reservoir hosts [2]. Among countries with areas endemic for schistosomiasis japonica, the Peoples’ Republic of China (P.R. China) had a very high disease burden in the 1950s with 11.6 million individuals and 1.2 million cattle infected with *S. japonicum* [3, 4].

High priority for schistosomiasis control is always given by the central government in P.R. China exemplified by the slogan of “Must eliminate schistosomiasis” issued in 1955 and poems of “Farewell to the God of Plague” written by the Chairman Mao Zedong [5]. Consecutive national programme was conducted with implementation of disease elimination strategy emphasized on snail control and later morbidity control strategy focused on chemotherapy, adapting to epidemiological insights, technological advances and economic status at that time before the new millennium [6, 7] . Although significant decrease of snail habitats and morbidity of schistosomiasis in residents and livestock occurred during 1992–2001 with the implementation of World Bank Loan Project for schistosomiasis control, infection and reinfection were unavoidable due to the continued transmission cycle of schistosomiasis [8].

The huge flooding along the Yangtze River that occurred in 1998 contributed to increasing numbers of infected people and further modulated by changes of economic, social and other factors at the beginning of twenty-first Century [3]. When this trend was confirmed, to protect human beings, a medium and long term national plan (MLNP) for schistosomiasis control was initiated in 2004, aiming at reaching morbidity control (both infection rates of schistosomes in residents and domestic animals less than 5%) by 2008 and transmission control (both infection rates of schistosomes in residents and domestic animals below 1%, and no infected snails detected in two consecutive years) by 2015 in all endemic villages, according to the criteria of schistosomiasis control and elimination defined by the Chinese government [6]. Obviously,the indicators to assess the stage of transmission control are much stricter than the threshold of eliminating schistosomiasis as a public health problem defined by WHO as the infection rate of high-intensity < 1% in all sentinel sites [9, 10]. Meanwhile, a comprehensive control strategy aiming to block the transmission of schistosomiasis was put forward through pilots first and then expanded in MLNP [6, 9].

To better understand the trends of schistosomiasis transmission and evaluate the effectiveness of the comprehensive control strategy, a systematic surveillance platform was constructed since 2005 [11] . Except for routine case reporting, the sentinel sites were enlarged from 20 to 80, covering more range and presenting all categories of endemic villages [12, 13]. In this article, we describe the changes of infection rates of schistosomes in human beings, domestic animals and snails, as well as the transmission features spanned the years from 2005 to 2015, using schistosomiasis sentinel surveillance data.

## Methods

### National sentinel surveillance sites

During 2005–2015, schistosomiasis endemic villages cross P.R. China were selected by modified stratified sampling method based on their eco-epidemiological features. In each provinces, the endemic areas were categorized by environmental ecosystems of snail infested area, including 1) lake and marshland region, 2) waterway networks in the plain region, 3) hilly and mountainous region [14]. Villages presenting local highest endemic situations were randomly selected as the sentinel sites, concerning the recent 3 years of epidemiological data. In phase of 2005–2010, 80 sentinel sites were distributed in nine endemic provinces and Chongqing City, the later regarded having the transmission potential of schistosomiasis due to the construction of the Three Gorges Dam. Due to the decreased compliance rate receiving examination and treatment, sentinel sites were adjusted in 2011 according to local situation. 69 sites remained the same during two phases, while other villages were adjusted. The number of sentinel sites accounted about 0.2% of total endemic villages nationwide. General information of selected sentinel sites including number of residents, ecological features, longitude and latitude of villages were collected and recorded.

### Surveillance of human population

Annual cross-sectional surveys were carried out in October or November in all sentinel villages after the transmission season. All residents aged 6 years or above were enrolled and a simple questionnaire was distributed to collect general demographic information. One blood sample over 250 μl was collected from each participant and centrifuged to separate sera. The obtained sera samples were tested by indirect hemagglutination assay (IHA, Anji Pharmaceutical Technology Co., Ltd) according to the manufacture’s protocol. The titer in the tested sera was recorded as one dilution before that which yielded a clear, sharp dark spot similar to that in the negative control wells. Titers were expressed as reciprocal values. Titers of ≥10 indicated a positive result [15].

At least 30 g of stool was collected from each IHA-positives and subjected to fecal examination. During 2005–2010, Kato–Katz thick smear technique (KK) was the only method to confirm an infection. While in 2011–2015, hatching technique (HT) was conducted in parallel with KK for one fecal sample to increase the detection rate [16, 17]. Briefly, three thick smear from each stool were prepared and read by experienced technicians. The number of schistosome eggs detected from each KK slide was recorded. The remaining fecal specimen was tested by miracidium HT. Observation was made at 4, 8, 12 h after hatching. Residents that were positive for KK and/or HT (egg positive and/or miracidium positive) were defined as parasitological positive.

### Animal surveillance

Domestic animals including bovines, pigs, goats, dogs, etc. raised in each sentinel sites were investigated simultaneously as the survey conducted on humans. The minimum sample size of each kind of domestic animals was 60 in each village (If the number of raised animals less than 60, all animals would be examined). Stools from domestic animals were collected and then examined annually by HT after peak transmission season [16]. The result can be defined as positive if miracidium was observed.

### Snail surveillance

The snail survey was conducted annually in surroundings where have or potentially have snails infested, by a square frame made by iron wire with 0.1 m^2^ area through a systematic sampling method combined with environmental sampling method [16]. All snails within the frames were collected, counted and brought to the laboratory to be examined in a dissecting microscopy to identify the infection status and whether they were alive or not. The snail was determined as infected one if the sporocyst and/or cercaria of schistosome was detected in the soft tissues of dissected snail.

### Data management and statistical analysis

Surveillance activities were conducted by Center for Disease Control (CDC) or Schistosomaisis Control Station at county level. Annual data reflecting the endemic situation of schistosomiasis were collected and entered into computer in a standardized manner and sent to National Institute of Parasitic Diseases, the Chinese Center for Disease Control and Prevention. Quantitative data were analyzed descriptively using the statistical software SPSS (version 13.0, SPSS Inc., Chicago, USA).

The trends of infection status of schistosomes were measured by infection rates of schistosomes in human residents, domestic animals and snails. Human infection rate in a sentinel village was calculated by the IHA positive rate (No. sero-positives/No. examined by IHA) times parasitological positive rate (No. positives in KK or HT/No. received stool examination) [18]. The number of eggs per gram feces (EPG) of infected residents determined by KK was calculated with the total number of eggs counted in three KK slides from one stool sample multiplying by eight. Infection rate of schistosomes in domestic animals was calculated by No. positives in HT/No. animals received stool examination [18]. For snails, infection rate of schistosomes in snails was determined as No. infected snails/No. dissected snails. Snail infested area, area with infected snails, area of new snail habitats were calculated according to the guidelines of schistosomiasis control handbook in China [16, 19]. The area reflecting the distribution of snails was expressed as length (meters) multiplied by width (meters). The length and width were determined based on the largest distance between two frames which found live snails or infected snails in the surveyed environments [19]. Confidence intervals (*CI*) were calculated using standard formulas based on the binomial distribution. ANOVA analysis was used to examine the changes of EPG in population and Chi-square test was used to examine any change in proportions over time. *P-*values less than 0.05 were considered to be statistically significant.

## Results

### General information of sentinel sites

**Fig. 1:**
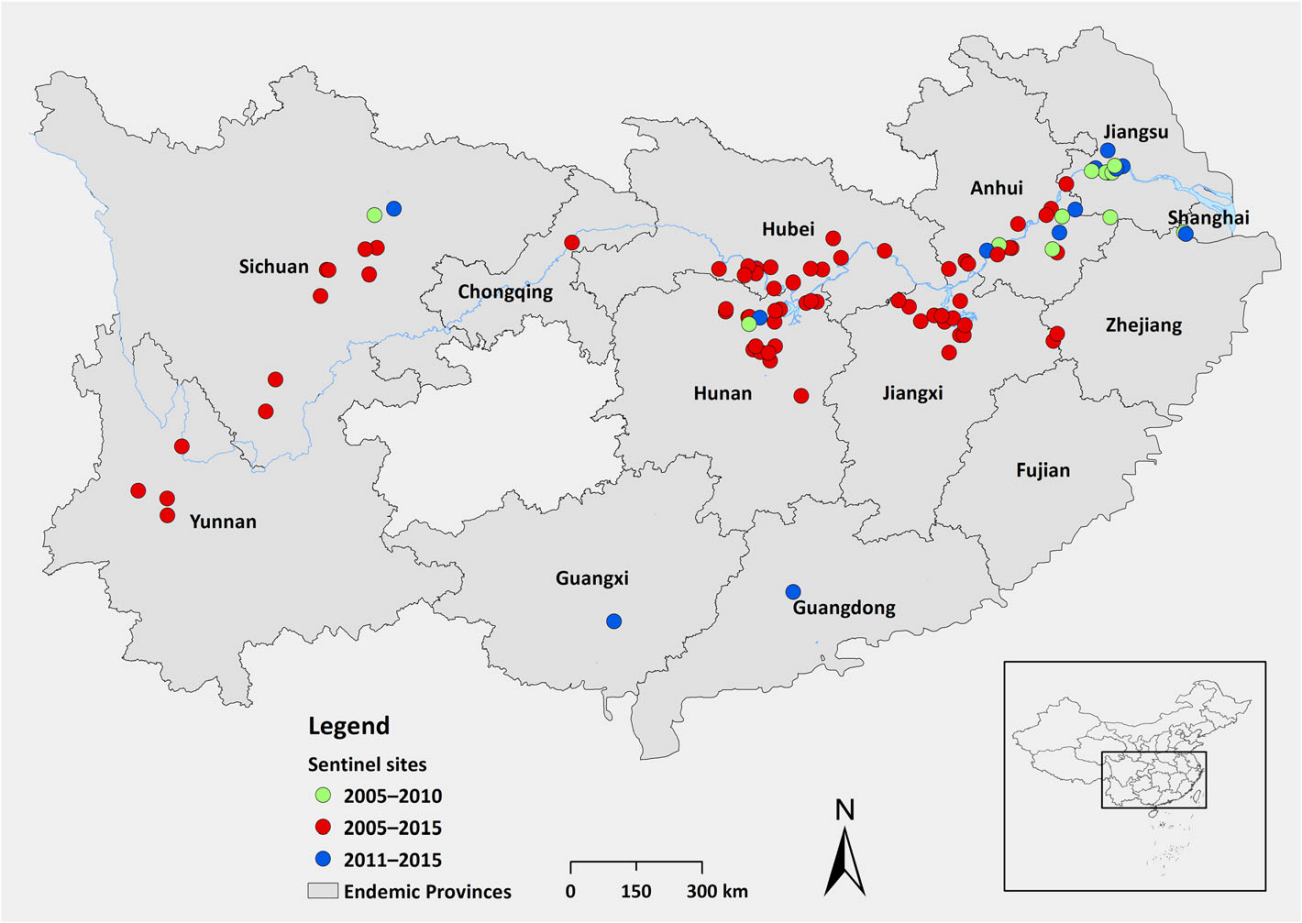
The geographical distribution of sentinel sites during 2005–2015

In phase of 2005–2010, among 80 sentinel sites, 53 were featured as lake and marshland regions, 23 were located in hilly and mountainous regions while the left four were located in waterway network in plain region, covering 130 703 residents. In second phase, one site characterized as waterway network in plain region was dropped from Jiangsu Province and one more each in Guangdong Province (waterway network in plain region) and Guangxi Zhuang Autonomous Region (hilly and mountainous region) were added respectively. The total number of the residents in sentinel sites in 2011 was 84 382. The geographical distribution of the sentinel sites is depicted in Fig. 1.

### Trends in residents

A total of 148 902 residents from sentinel sites attended this study and 631 676 blood samples were examined by IHA test during the 11 covered years. The ratio of males to females was 0.96∶1 (72 689 males/75 922 females), while gender information of 291 blood providers was missed. The annual average antibody positive rates presented a significant decrease trends, from 17.48% (95% *CI*: 17.20–17.75%) in 2005 to 5.93% (95% *CI*: 5.71–6.15%) (*χ*^2^ = 8890.47, *P* < 0.001) in 2015 (Table 1).

**Table 1:** Changes of infection rates of schistosomes in population during 2005–2015 in sentinel sites

Phase	Year	IHA test			No. stool samples received examination^a^	Kato-Katz		No. HT positives	No. parasitological positives	Infection rate (%, 95 *CI*)
		No. sera samples examined	No. positives	Antibody positive rate, % (95% *CI*)		No. KK positives	Arithmetic mean EPG (mean ± SD)			
I	2005	72 595	12 686	17.48 (17.20–17.75)	12 226	1447	12.37 ± 124.62	–	1447	2.07 (1.96–2.17)
	2006	64 336	8770	13.63 (13.37–13.90)	8406	961	10.95 ± 88.34	–	961	1.56 (1.46–1.65)
	2007	60 535	7484	12.36 (12.10–12.63)	7215	537	3.16 ± 22.88	–	537	0.92 (0.84–1.00)
	2008	63 202	6670	10.55 (10.31–10.79)	6104	416	3.03 ± 25.44	–	416	0.72 (0.65–0.79)
	2009	62 039	5523	8.90 (8.68–9.13)	5293	304	2.71 ± 22.89	–	304	0.51 (0.46–0.57)
	2010	59 311	5636	9.50 (9.27–9.74)	5358	314	4.93 ± 143.59	–	314	0.56 (0.50–0.62)
II	2011	54 550	4249	7.79 (7.56–8.01)	4155	194	1.94 ± 16.21	222	289	0.54 (0.48–0.60)
	2012	53 040	3869	7.29 (7.07–7.52)	3759	136	1.47 ± 14.76	102	173	0.34 (0.29–0.38)
	2013	50 648	3349	6.61 (6.4.40–6.83)	3163	75	0.70 ± 6.54	95	112	0.23 (0.19–0.28)
	2014	47 457	2858	6.02 (5.81–6.24)	2802	32	0.41 ± 6.38	42	48	0.10 (0.07–0.13)
	2015	43 963	2608	5.93 (5.71–6.15)	2600	36	1.14 ± 14.96	46	55	0.13 (0.09–0.16)
Total		631 676	63 702	–	61 081	4452	–	507	4656	–

^a^only done in individuals with positive IHA results

*IHA* Indirect hemagglutination assay, *KK* Kato-katz, *EPG* Eggs per gram feces, *HT* Hatching technique, *CI* confidence intervals

Among 63 702 antibody positives detected during 2005–2015, 95.89% (61 081/63 702) received stool examination. By KK method, the number of egg positives was decreased from 1447 in 2005 to 36 in 2015, with mean EPG decreased significantly from 12.37 ± 124.62 to 1.14 ± 14.96 (F = 2.30, *P* = 0.01). In 2015, only one infected resident had EPG of 512, in the range of high infection intensity. During 2011–2015, HT was conducted in parallel with KK method, with 507 positives and 473 positives totally detected by each method. Combined the results of KK and MH methods, totally 4656 of stool samples were determined as parasitological positives during 2005–2015, with a significant declined trend (Table 1).

**Fig. 2:**
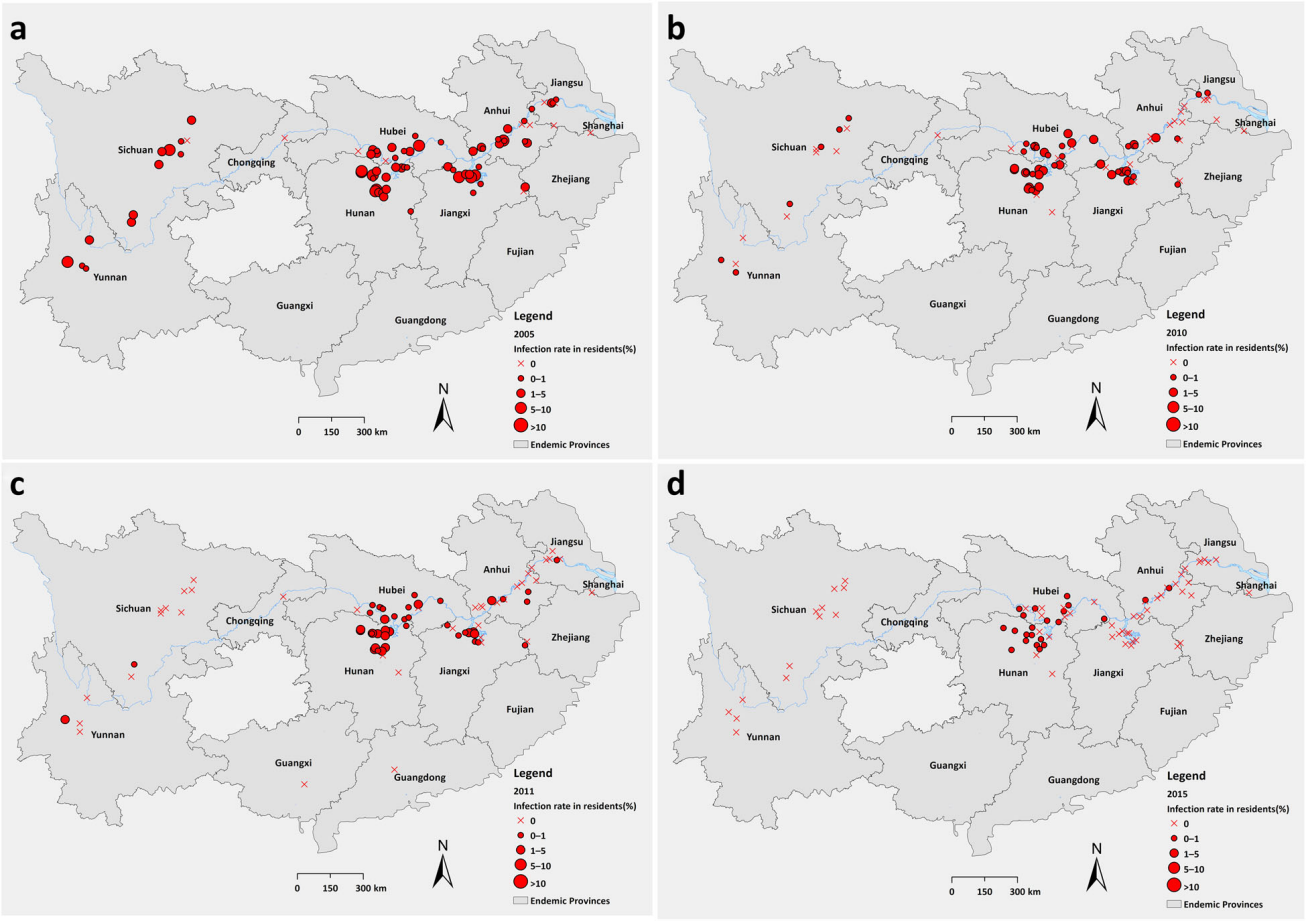
Changes of infection rates of schistosomes in residents from 2005 to 2015 based on village level. **a**: 2005; **b**: 2010; **c**: 2011; **d**: 2015

In combination of the result of serological test and parasitological tests, the infection rate of schistosomes in residents declined from 2.07% (95% *CI*: 1.96–2.17%) in 2005 to 0.13% (95% *CI*: 0.09–0.16%) in 2015 at a high level of statistical significance (*χ*^2^ = 751.24, *P* < 0.001) (Table 1). In 2005, 1447 stool positives distributed in 86.25% (69/80) sentinel villages, while 55 stool positives in 2015 were found in 27.16% (22/81) villages. Based on village level, the highest infection rate was 13.34% in 2005 and 0.87% in 2015, both from the lake and marshland regions of Hunan Province (Fig. 2).

**Fig. 3:**
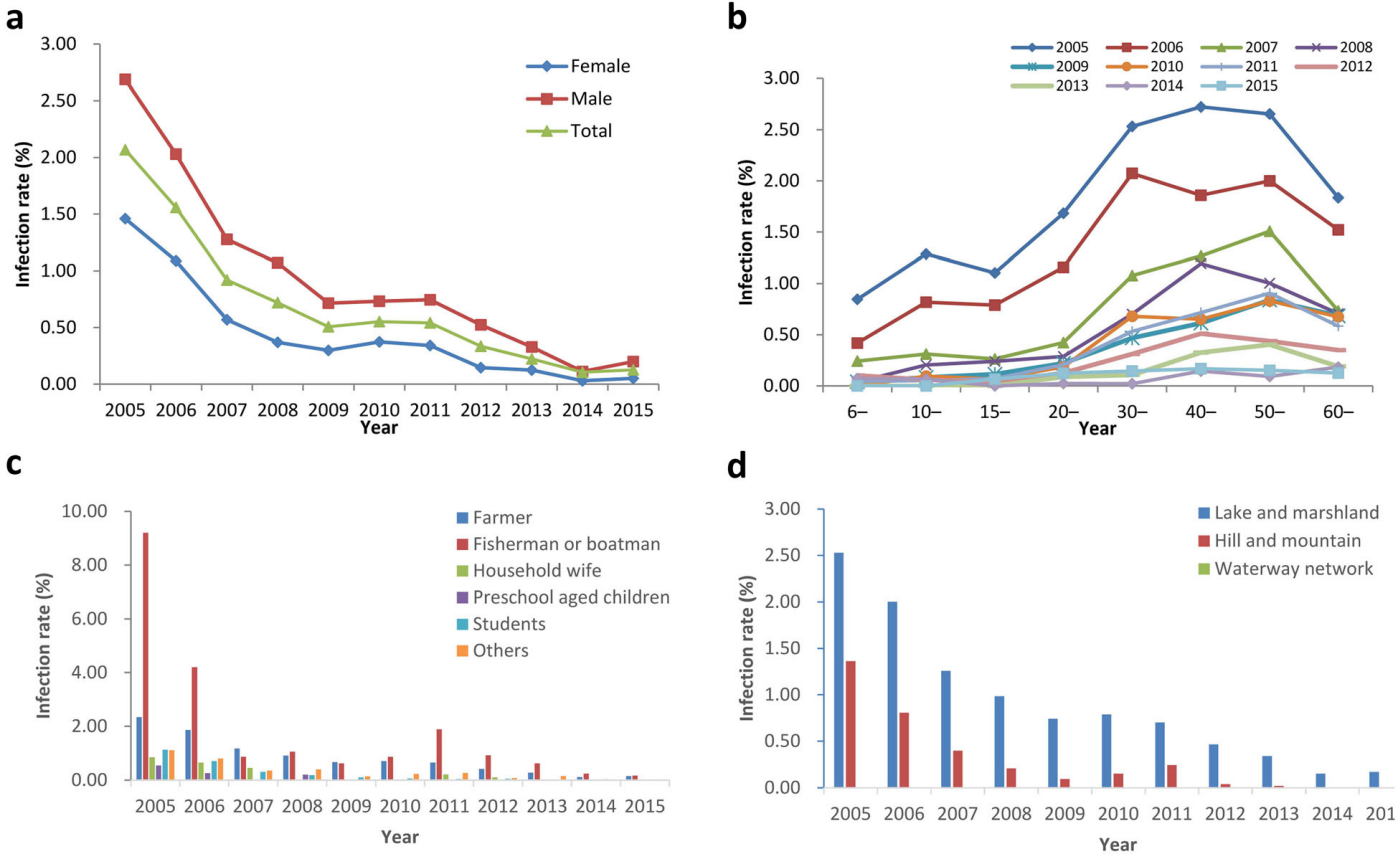
Changes of infection rates of schistosomes in residents from 2005 to 2015 by strata. **a**: by gender; **b**: by age; **c**: by occupation; **d**: by eco-epidemiology

The infection rates of schistosomes in residents classified by gender, age, occupation, eco-epidemiological types were shown in Fig. 3. The infection rate in males was decreased from 2.69% (95% *CI*: 2.52–2.85%) in 2005 to 0.20% (95% *CI*: 0.14–0.26%) in 2015, and in females decreased from 1.46% (95% *CI*: 1.34–1.58%) to 0.05% (95% *CI*: 0.02–0.08%). Males generally presented a higher infection rate than the females during 2005–2015 (Fig. 3a). The infection rate of schistosomes in residents peaked in the elder age group and decreased with time went by (Fig. 3b). The population group aged 40–49, 50–59 years old presented the highest infection rate of 2.72% (95% *CI*: 2.48–3.06%), 2.66% (95% *CI*: 2.33–2.93%) in 2005 and decreased to 0.15% (95% *CI*: 0.07–0.22%), 0.17% (95% *CI*: 0.09–0.25%) in 2015 respectively. Analyzed by occupation, fishermen and boatmen, and farmers were the major population infected with schistosomes with the highest infection rates almost each year (Fig. 3c). In 2015, the stool positives were only found in farmers, fishermen and boatmen with infection rate of 0.16% (95% *CI*: 0.11–0.20%), 0.17% (95% *CI*: 0–0.50%) respectively. By eco-endemic types of sentinel sites, lake and marshland regions always preformed the highest infection rate, but also decreased significantly from 2.53% (95% *CI*: 2.39–2.67%) in 2005 to 0.17% (95% *CI*: 0.13–0.22%) in 2015, while the infection rate of schistosomiasis in hilly and mountainous regions reached zero since 2014 and no infection was found in residents from water-way network regions since 2005 (Fig. 3d).

### Trends in domestic animals

**Fig. 4:**
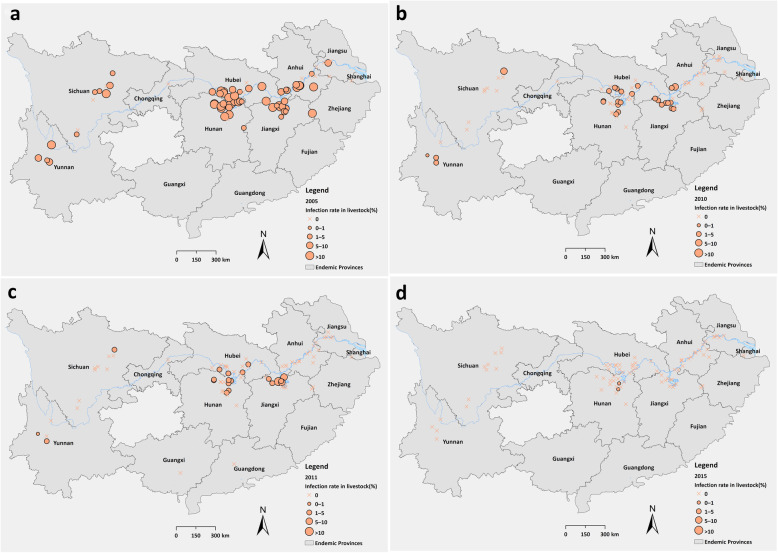
Changes of infection rates of schistosomes in domestic animals from 2005 to 2015 based on village level. **a**: 2005; **b**: 2010; **c**: 2011; **d**: 2015

During 2005–2015, total 37 822 domestic animals received stool examination by HT and 1203 positives were determined. The average infection rate of schistosomes in domestic animals declined from 9.42% (538/5711, 95% *CI*: 8.66–10.18%) in 2005 to 0.08% (2/2360, 95% *CI*: 0–0.19%) in 2015 significantly (*χ*^2^ = 233.13, *P* < 0.001) (Table 2). In 2005, infected domestic animals were found in 57 villages among 72 villages where had livestock raised. The highest infection rate reached 65.91% (29/44, 95% *CI*: 51.90–79.92%) in a sentinel site within Yueyang City of Hunan Province. In 2015, there were only 44 villages raising domestic animals and only two infected cattle were detected in two separate sentinel sites of Hunan Province (Fig. 4).

**Table 2:** Changes of infection rates of schistosomes in domestic animals during 2005–2015 in sentinel sites

Phase	Year	No. examined	No. positives	Infection rate, % (95% *CI*)
I	2005	5711	538	9.42 (8.66–10.18)
	2006	5365	318	5.93 (5.30–6.56)
	2007	3693	107	2.90 (2.36–3.44)
	2008	3486	58	1.66 (1.24–2.09)
	2009	3538	65	1.84 (1.39–2.28)
	2010	3225	53	1.64 (1.20–2.08)
II	2011	3712	36	0.97 (0.65–1.29)
	2012	2670	20	0.75 (0.42–1.08)
	2013	1969	5	0.25 (0.03–0.48)
	2014	2093	1	0.05 (0–0.14)
	2015	2360	2	0.08 (0–0.20)
Total		37 822	1203	–

**Fig. 5:**
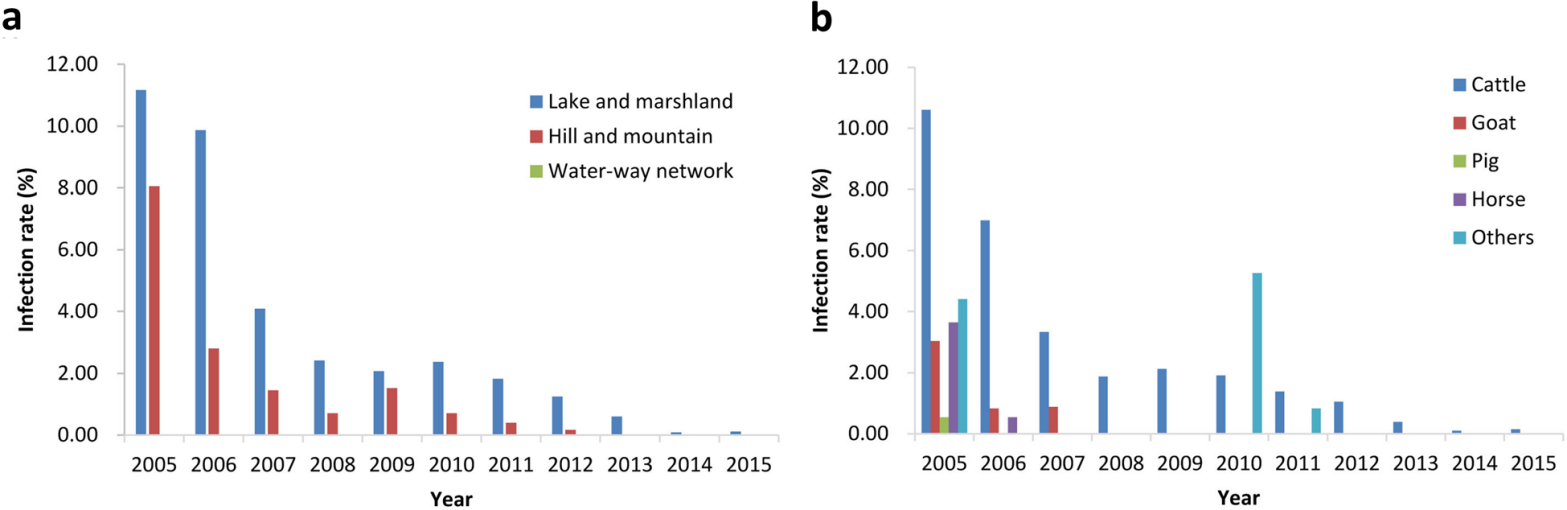
Changes of infection rates of schistosomes in domestic animals by eco-epidemiological and species strata. **a**: by eco-epidemiology **b**. by species strata

At the beginning of the sentinel surveillance, the infection rate of schistosomes in domestic animals was very high both in lake and marshland regions, hilly and mountainous regions, with the value of 11.17% (300/2685, 95% *CI*: 9.98–12.36%) and 8.05% (238/2955, 95% *CI*: 7.07–9.03%) in 2005 respectively. However, the infection rates of schistosomes in domestic animals decreased zero in hilly and mountainous regions since 2013. And no infection of schistosomes was found in domestic animals in four sentinel sites featured by waterway network in plain regions during 2005–2015 (Fig. 5a). In 2005, the highest infection rate of schistosomiasis in cattle was 10.61% (515/4853, 95% *CI*: 9.74–11.48%), and positives were also found in other seven species including goat, pig, horses etc. with infection rates in the range of 0.55–4.11%. Since 2012, infections were only detected in cattle (Fig. 5b).

### Trends in snails

Snail investigation was conducted in 79 351 ha of areas cumulatively during 2005–2015. Totally 4 119 055 frames were set for snail’s investigation and 1 350 028 living snails were found in 350 712 set frames. By dissection method, totally 2408 infected snails were found. The area of snails’ habitats varied from 5150 ha to 3859 ha in a decreased trend as time went by. Totally 98 ha of new snail habitats were found in the period of 2005–2010, while 94.90% (93/98) distributed in lake and marshland regions. The area with infected snails decreased from 482 ha in 2005 to zero since 2014 in all sentinel sites.

**Fig. 6:**
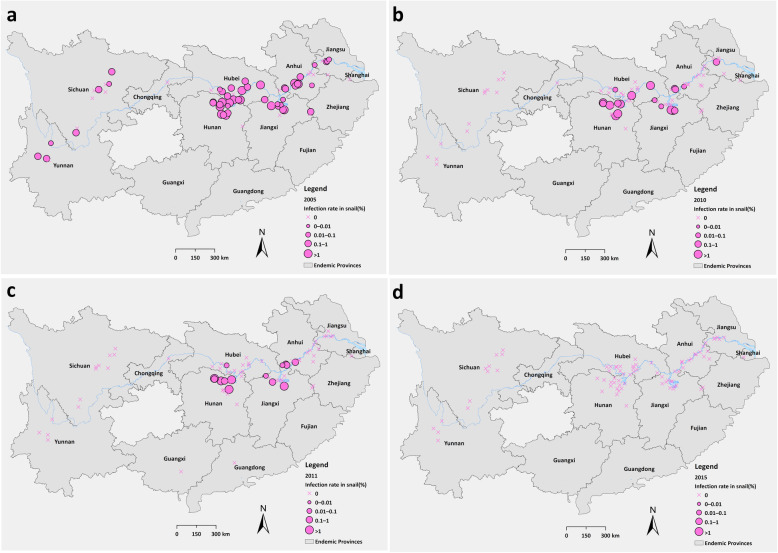
Infection rates of schisotosomes in snails based on village level. **a**: 2005; **b**: 2010; **c**: 2011; **d**: 2015

The percentage of frames infested with snails decreased from 16.96% (56 884/335391, 95% *CI*: 16.83–17.09%) in 2005 to 4.28% (18 121/423755, 95% *CI*: 4.22–4.34%) in 2014, with a slightly increase in 2015. Meanwhile, the infection rate of schistosomes in snails was decreased year by year significantly with *P* < 0.001 (Table 3). In 2005, among 50 villages found infected snails, 40 distributed in lake and marshland regions while 10 in hilly and mountainous regions, with the highest infection rate of 14.04% (8/57, 95% *CI*: 5.02–23.06%) occurred in a village from Hubei province. There was no positive snail found through traditional dissection method in 2014 and 2015 (Fig. 6).

**Table 3:** Results of snail survey during 2005–2015 in sentinel sites

Surveillance phase	Year	No. area surveyed (ha)	No. area with infested snails (ha)	No. area of new snail habitat(ha)	No. area with infected snails(ha)	No. frames surveyed	No. frames with snails	No. dissected living snails	No. infected snails	Percentage of frames with living snails, % (95% ***CI***)	Infection rate, % (95% ***CI***)
I	2005	6966	5150	13	482	335 391	56 884	256 531	663	16.96 (16.83–17.09)	0.2584 (0.2388–0.2781)
	2006	7462	5014	22	625	374 149	47 808	203 828	744	12.78 (12.67–12.88)	0.3650 (0.3388–0.3912)
	2007	7110	4603	1	244	356 623	41 927	152 389	297	11.76 (11.65–11.86)	0.1949 (0.1728–0.2170)
	2008	7372	4633	8	176	362 537	32 455	117 615	178	8.95 (8.86–9.05)	0.1513 (0.1291–0.1736)
	2009	7395	4570	0	134	360 932	31 964	121 574	214	8.86 (8.76–8.95)	0.1760 (0.1525–0.1996)
	2010	7087	4294	54	173	380 727	29 666	106 107	187	7.79 (7.71–7.88)	0.1762 (0.1510–0.2015)
II	2011	7009	4037	0	98	376 348	24 309	83 775	117	6.46 (6.38–6.54)	0.1397 (0.1144–0.1649)
	2012	6974	3859	0	4	398 717	20 748	73 979	7	5.20 (5.13–5.27)	0.0095 (0.0025–0.0165)
	2013	7157	4093	0	1	356 279	18 033	54 091	1	5.06 (4.99–5.13)	0.0018 (0.0000–0.0055)
	2014	7248	4100	0	0	423 755	18 121	66 522	0	4.28 (4.22–4.34)	0.0000
	2015	7571	4147	0	0	393 597	28 797	11 617	0	7.32 (7.24–7.40)	0.0000
Total		79 351	48 500	98	1937	4 119 055	350 712	1 350 028	2408	–	–

*ha* hectare, *CI* Confidence intervals

## Discussion

Schistosomiasis is a water borne communicable disease and mainly endemic in the poor and rural areas of the world, regarded as a disease difficult to be controlled. Due to the reduction of the cost of praziquantel, an effective drug for treatment, and increased funding for schistosomiasis control programmes in recent decades, the number of people receiving preventive chemotherapy with praziquantel increased worldwide [20]. Although the argument on the goal set for schistosomiasis elimination continues [21, 22], opinions tend to be consistent that schistosomiasis is one of diseases being potentially eliminated in countries with low endemicity, adequate resources and political commitments provided [23, 24]. With the implementation of the twelve-year’s MLNP in P.R. China, the annually national reports showed that the number of estimated cases had been dramatically decreased from 798 762 in 2005 to 77 194 in 2015, with a reduction rate of 90.34% [25, 26]. The results of our sentinel surveillance data in this article provided evidence that the infection and transmission risk of schistosomiasis was reduced significantly in P.R. China. It is feasible to eliminate schistosomiasis as a public health problem at a country level and transmission interruption could be reached at a regional or country level after application of intensive and comprehensive approaches.

Measurement of the changes of infection rates of schistosomes in humans, livestock and snails could provide the best indication of the effectiveness of the interventions on schistosomiasis. The national sentinel surveillance conducted in 2005–2015 in P.R. China illustrated significant decline of transmission intensity and changes of transmission pattern due to continued interventions against schistosomiasis. The infection rate of schistosomes in residents decreased by more than 90% from 2005 to 2015. The decreased and delayed peak of infection rate in residents with age especially in 2015 may due to the lower transmission risk in living surroundings as fewer infected domestic animal and no infected snails detected, higher knowledge level towards schistosomiasis control and prevention in school-aged children or young adults by health education, or the changed population structure in rural areas as more young and mid-age adult moving to city over the past decades [27]. Fishermen and boatmen were the major population groups responsible for a large proportion of environmental contamination with *Schistosoma* eggs, due to their frequent water contact behavior [28]. The administration of chemotherapy or stool collection for these groups is very difficult due to their floating behavior and continuous water contact [29, 30] . As there are more than 10 thousand fishermen and boatmen in the Poyang and Dongting Lake, respectively, new approaches targeted for this groups for blocking transmission are needed urgently when the national schistosomiasis programme moving towards elimination stage.

Schistosomiasis japonica is a zoonotic disease, as more than 40 species of domestic and wild animals acting as reservoir hosts of *S. japonicum* [31]. Chemotherapy was the major intervention against animal infection in P.R. China before 2004. Evidence showed that this approach could decrease the infection rate and morbidity of schistosomiasis in livestock to a relative low level, but couldn’t prevent reinfection, particularly in the lake and marshland region [32]. With the implementation of integrated control measures, the infection rate of schistosomiasis in domestic animals declined by 99%, and only two infected cattle detected in two villages of Hunan Province in 2015. Meanwhile, various domestic animals were detected stool positive at the beginning of surveillance while cattle always presented the higher positive rate according to the surveillance, which consistent with former studies proving bovines’ role in the transmission of schistosomiasis [33, 34]. However, some studies investigated that dogs, goats or wild mice still played important roles on schistosomiasis transmission in some certain endemic areas where transmission of schistosomiasis had been under control or there were no domestic animal raised [35–37]. Understanding the major species of domestic and wild animals serving as reservoir hosts in local settings timely thus to take relevant countermeasures, are necessary to consolidate the achievements obtained and facilitate the progress towards schistosomiasis elimination.

Although snail control was neglected in most schistosomiasis endemic countries, it is an important intervention to interrupt the life cycle of *Schistosoma* to reduce the endemic range and transmission risk in P.R. China [6, 38, 39]. Through snail control approach, the area of snail habitats had decreased from about 1.43 million hectare in the 1950s to 0.38 million hectare by 2003 before the MLNP [3]. In our study, the decline of snail habitats was slowly and slightly from 2005 to 2015 in the sentinel sites, but the areas of infected snails, the density of snails and infection rate of schistosomes in snails were significantly decreased, thus lowered the risk of schistosomiasis transmission [40]. The recent publication also demonstrated snail control is an important component of the national schistosomiasis control strategy [41]. In addition, a total of 98 ha of new snail habitats had been explored in sentienl sites during 2005–2015, which mostly distributed in the lake and marshland regions, proved once again the difficulty in eliminating an amphibious species-*Oncomelania hupensis*, but we are able to eliminate the infected snails [27, 40, 42, 43] .

Based on the evidence obtained from the sentinel sites, in combination with the annual data obtained from endemic provinces, P.R. China has reached the final goal set by the MLNP to reach the transmission control, also eliminate schistosomiasis as a public health problem one year earlier than the deadline settled by WHO Western Pacific Region Office [6, 44, 45], and it is also only one country reached this goal by 2020 in Western Pacific Region. The strategic plan of Healthy China 2030 issued by Chinese government set the goal of eliminating schistosomiasis nationwide by 2030 [46]. To reach the final goal of schistosomiasis elimination, mechanisms of multi-sector cooperation and government leader role should be insisted to ensure the available resources for schistosomiasis control efforts in all endemic regions. During the transition stage from control to elimination, effective control activities should be conducted more precisely due to the changes of transmission pattern and heterogenous endemicity of schistosomiasis, supported by the precise tools or technologies [47, 48].

Contradictory with the decreased trend of infection rates based on sentinel surveillance data, we also noticed that challenges existed to eliminate schistosomiasis nationwide especially in the areas around the Dongting Lake and Poyang Lake, due to the large areas of snail habitats, newly developed snails infested areas, large amount of fishermen and boatmen, various animal reservoir hosts etc. As elimination of a disease is defined as reducing a locally acquired infection to zero incidence in a specific, geographic area through deliberate efforts, surveillance and response systems appear to be the most cost-effective way to improve the efficiency in the disease elimination [49, 50]. As surveillance has been major important intervention in the national schistosomiasis elimination program in P.R. China, surveillance-response systems should be further improved and strengthened through developing and implementing effective and precise tools for surveillance, owning a well-trained team for case finding, data reporting, foci tracking and rapid action, improving the accuracy and timeliness of data collection, analysis, interpretation and early warning systems, tailoring precise surveillance activities into local situations. The sensitive and effective surveillance-response systems will not only promote the process of schistosomiasis towards elimination, but also provide scientific evidence for schistosomiasis elimination programme in P.R. China.

Several limitations of this study should be noted. One is the adjustment of sentinel sites in the year of 2011, by which the surveillance data may result in a biased sample and participants although the eco-epidemiological feature and numbers of sentinel sites from each province are almost the same between two phases. The other one is the representation of the residents participated in the investigation, in which the population structure and movement were not considered as biased factors in the data analysis, so the infection rate we obtained is the crude value that only can used for observing the change patterns. In addition, only four or five sentinel sites were selected from provinces where transmission had been interrupted, the average infection rate based on the sentinel surveillance data was overestimated compared to the real value at national level.

## Conclusions

Eleven years of surveillance data presented the decreased trends of infection rates of schistosomes in residents, domestic animals and snails, with the implementation of the integrated control strategy. Elimination of schistosomiasis as a public health problem was reached in P.R. China nationwide according to WHO’s definition. With the changes of transmission features and existed challenges for schistosomiasis elimination, surveillance-response systems should be further improved and strengthened in P.R. China in order to achieve the goal of schistosomiasis elimination by 2030.

## Data Availability

All data generated or analyzed during this study are confidentially kept at the National Institute of Parasitic Disease, Chinese Center for Disease Control and Prevention. The datasets are available from the corresponding author on a reasonable request.

## Abbreviations

CDC: Center for Disease Control
CI: Confidence intervals
EPG: Eggs per gram feces
HT: Hatching technique
IHA: Indirect hemagglutination assay
KK: Kato-katz method
MLNP: The Medium and long term national plan
P.R. China: People’s Republic of China
WHO: World Health Organization
